# Influence of menstrual cycle, parity and oral contraceptive use on steroid hormone receptors in normal breast.

**DOI:** 10.1038/bjc.1992.122

**Published:** 1992-04

**Authors:** S. Battersby, B. J. Robertson, T. J. Anderson, R. J. King, K. McPherson

**Affiliations:** Department of Pathology, University Medical School, Edinburgh, UK.

## Abstract

**Images:**


Br. J. Cancer (1992), 65, 601–607 © Macmillan Press Ltd., 1992

### Influence of menstrual cycle, parity and oral contraceptive use on steroid hormone receptors in normal breast

S. Battersby[1], B.J. Robertson[1], T.J. Anderson[1], R.J.B. King[2] & K. McPherson[3]

[1]*Department of Pathology, University Medical School, Edinburgh;* [2]*ICRF Breast Biology Group, School of Biological Sciences, University of Surrey, Guildford and* [3]*Department of Public Health and Policy, London School of Hygiene and Tropical Medicine, London, UK.*

**Summary** Steroid receptor was assessed immunohistochemically in 158 samples of normal breast for variation through the menstrual cycle. Patterns and intensity of reaction were used in a semi-quantitative scoring system to examine the influence of cycle phase, cycle type, parity and age. The changes in oestrogen receptor for natural cycle and oral contraceptive (OC) cycles indicated down-regulation by progestins. Progesterone receptor did not vary significantly in natural cycles, but increased steadily through OC cycles. This study provides strong evidence that both oestrogen and progesterone influence breast epithelium, but dissimilarities from the endometrium are apparent. The interval since pregnancy had a significant negative effect on frequency and score of oestrogen receptor and score of progesterone receptor. Multivariate analysis established the phase of cycle and OC use as independent significant influences on oestrogen receptor. The interval since pregnancy was an independent significant factor for both oestrogen and progesterone receptor presence.

Female sex steroids, oestrogens and progestins, influence the normal human breast, but controversy exists as to the nature of those effects, especially in relation to cell proliferation (McCarty, 1989; King, 1990). In part, the controversy reflects the difference in data derived from tissues obtained *ex vivo* from those derived from cell culture and from xenografts in nude mice, but another factor has been the assumption of similarity in the steroidal regulation of breast and endometrial epithelia. While a clear picture of oestrogen receptor and progesterone receptor regulation exists for endometrium (Lessey *et al.*, 1988), the same is not true for breast. Oestrogen and progesterone receptors (OR and PR) are both present in normal human breast epithelium (Petersen *et al.*, 1987; Jacquemier *et al.*, 1990; Joyeux *et al.*, 1990; Williams *et al.*, 1991) but with lack of agreement between authors as to differences across the cycle or consistency within it. To help clarify the situation, we have carried out an immunohistochemical analysis of OR and PR in histologically normal breast tissue, related the findings to phase of the menstrual cycle, oral contraceptive (OC) use, age and parity. These factors have been demonstrated previously to have a major effect on breast proliferation (Anderson *et al.*, 1989). Information on the influence of OC on breast OR and PR is relevant, in view of the importance of this topic in relation to the genesis of breast cancer (Thomas, 1991). This report addresses these issues and includes a morphological evaluation of the patterns and distribution of receptor staining within the breast parenchyma.

### Materials and methods

Breast tissue was obtained from 158 premenopausal women undergoing biopsy for clinical reasons, as described previously (Anderson *et al.*, 1989). Samples of histologically normal tissue were taken from areas adjacent to fibroadenomas (62%), minimal fibrocystic change (14%) or from specimens in which no significant histopathology was identified (24%). Menstrual cycle and obstetric history were obtained on the day of biopsy and menstrual cycle length identified by postal returns was normalised to 28 days (Anderson *et al.*, 1989). The menstrual cycle was divided into 'weeks' of unequal length, to take account of days on and off OC, as described previously (Anderson *et al.*, 1989).

Correspondence: Dr T.J. Anderson, Department of Pathology, University of Edinburgh, Medical School, Teviot Place, Edinburgh EH8 9AG, UK.
Received 4 April 1991; and in revised form 7 November 1991.

### *Tissue preparation*

Fresh biopsy specimens were transported to the laboratory on ice and areas of normal breast tissue (10 mm × 10 mm × 3 mm) were snap frozen in isopentane in liquid nitrogen and stored at −80°C until use. Adjacent areas were fixed in methacarn fixative (methanol: chloroform: acetic acid, 6:3:1) at 4°C and processed to paraffin wax.

For immunohistochemical localisation of OR and PR, cryostat sections were obtained and fixed in Zamboni's fixative (Stefanini *et al.*, 1967) for 15 min at −20°C and stored in glycerol/sucrose buffer (Abbott ER-ICA handbook) at −20°C until use. Sections of methacarn fixed, paraffin embedded material were cut at 4 μm and low temperature dried.

### *Immunohistochemistry*

Oestrogen receptor localisation was performed using the ER-ICA rat monoclonal antibody (H222) to the OR (Abbott). Staining was performed either on frozen sections using the ER-ICA kit reagents or on methacarn fixed paraffin sections, using a modification of the method of Shintaku and Said (1987), previously shown to give comparable results to frozen sections in breast carcinomas (Paterson *et al.*, 1990). Briefly, dewaxed sections were treated with DNAase, followed by overnight incubation at 4°C with the primary antibody. After washing, sections were incubated sequentially with biotinylated goat anti-rat immunoglobulins (Sigma) and a streptavidin-biotin peroxidase complex (Amersham). The reaction was visualised with diaminobenzidine. Progesterone receptor was localised on cryostat sections using a mouse monoclonal antibody raised against rabbit uterine PR (Transbio, Paris), shown to exhibit a high affinity against the human PR (Helin *et al.*, 1988). The antibody was used at a dilution of 10 μg $ml^{-1}$ and detection was by an indirect immunoperoxidase method (Dako). An Abbott ER-ICA control section and a section of a breast carcinoma with a high level of PR, as determined biochemically, were included in each experiment as positive controls for OR and PR respectively.

### *Scoring*

One tissue section was examined in each case, and all terminal duct lobular units (TDLU) present were assessed (range = 6–78, mean 29, for paraffin sections and range = 5–29, mean 11, for frozen sections). Initially cases were scored on a simple positive or negative basis for dark brown chromogen reactivity, consisting of a minimum of 5% of nuclei in any parenchymal structure. Staining within the extralobular portion of the terminal duct (ELTD) was also evaluated, but was not included in the score. From the preliminary qualitative assessment of immunostaining, a semi-quantitative scoring system was developed to take account of three major variables considered to be important. These were: (1) the intensity of staining, (2) the percentage of TDLU showing reactivity, and (3) the morphological pattern of reactivity within the TDLU. If less than 5% of cells in a TDLU were reactive, it was considered as negatively stained. Intensity of reactivity was divided into three grades, where 1 = weak or equivocal, 2 = moderate and 3 = strong. The pattern of reactivity was graded as 1 if staining within a TDLU was of the sporadic type and 2 if greater than 30% of cells were positive, usually corresponding to a 'ring' of positive reacting cells within ductules. The score was calculated using a modification of a previously published summation equation (McCarty *et al.*, 1985).

$$\text{Score} = \sum \quad Pip\ (i + p + 1)$$

$Pip$ = percentage of TDLU stained for each pattern and intensity.
where $i$ = intensity of staining = 1, 2 or 3
$p$ = pattern of staining = 1 (sporadic) or 2 ('ring-like')

Non-reactive TDLU, while contributing to the calculation of the percentage of positively staining units in the overall summation, were given a score of 0. As an illustration, taking a case with 5 TDLU with negative staining, 5 of sporadic pattern at intensity 2, 5 of sporadic pattern at intensity 3 and 10 with a 'ring-like' pattern at intensity 1 results in a score of

$$20 \times 0 + 20(2 + 1 + 1) + 20(3 + 1 + 1) + 40(1 + 2 + 1) = 340$$

Twenty-five cases were examined independently by two observers and the scores obtained showed a significant concordance ($\tau = 0.723$, $P = 0.00003$, Kendall Rank Correlation). Intraobserver correlation was also highly significant ($\tau = 0.96$, $P = 0.00007$).

### *Statistical methods*

For univariate analysis the chi squared ($\chi^2$) test of significance was applied to the postive/negative scored data set out in contingency tables. Semi-quantitative scores were compared using the Mann-Whitney $U$ test with corrections made for ties. Multiple logistic regression and multiple regression were used with GLIM statistical software (NAC Computing Oxford, England).

### Results

### *Location and patterns of steroid receptor staining*

Immunohistochemistry for OR was performed in 158 cases. Staining was undertaken either on cryostat sections only (48), on paraffin sections only (63) or by both methods (47). OR staining was confined to luminal and intermediate epithelial cell nuclei and a highly significant concordance ($P < 0.001$) was seen, comparing staining on paraffin and cryostat sections. However, in five cases, positive reactivity was seen on paraffin sections, but not on cryostat sections from adjacent areas of breast tissue. The positive methacarn value was accepted, because the paraffin section gave a larger sample with a validated technique. Cases which were evaluated only on cryostat sections were equally distributed amongst various groups, e.g. OC users, nulliparous.

A total of 133 of the cases used above were stained for PR, using cryostat sections only. Steroid receptor detection was always nuclear in location and although predominantly in the luminal epithelial component of the TDLU, reactivity of the basal myoepithelial cells was also occasionally noted. In a low proportion of those cases where adjacent cryostat sections were stained for OR and PR particular TDLU were positively reactive for both antigens. In these cases staining was frequently of the 'ring-like' pattern described below.

The distribution of receptor staining for both steroids was heterogeneous within the tissue sample and in more than 80% of positive cases both positively and negatively stained TDLUs were seen on sections examined. In a few cases only one TDLU showed reactivity. Both positive and negative receptor reactivity was seen in the ELTD, with a positively reactive duct usually associated with at least one positively staining TDLU. Staining showed two morphological patterns; a sporadic pattern, where 5–30% of nuclei within a TDLU were reactive (Figure 1) or a 'ring-like' pattern, where more than 30% of nuclei showed positive staining (Figure 2). Although initial examination suggested that the 'ring-like' pattern was often intensely staining, formal analysis gave no significant relationship between the pattern and intensity of staining. Figures 1 and 2 specifically illustrate PR staining, but patterns of OR staining were very similar. Appreciation of the variations in staining pattern led to the development of a semi-quantitative scoring system which took pattern of reactivity into account as well as the more commonly assessed parameters of staining intensity and frequency.

The results (Tables I and II) were set down in specific case groupings, with receptor immunoreactivity assessed in two ways, either (1) the proportion of cases reactive, or (2) receptor scores. As regards the first, OR reactivity was present in 60 out of 158 (38%) of cases, while 96 out of 133 (72%) of cases were PR positive. Considering the second, OR scores ranged from 0 to 513 (median = 0), while for PR a range of 0 to 600 (median = 186) was seen. The following sections deal with aspects of staining for OR and PR, examining specifically the effects of menstrual cycle phase and type (i.e. natural or OC) as well as considering the influence of age and parity.

### *Oestrogen receptor detection*

*Effect of menstrual cycle type and day* In the natural cycle, 47% of cases showed positive immunoreactivity for the OR.

**Figure 1** Sporadic pattern of progesterone receptor immunoreactivity in epithelial nuclei of breast TDLU, ×320.

**Figure 2** 'Ring-like' progesterone receptor staining of luminal epithelial cell nuclei in normal breast, ×320.

When cases were assessed on a simple positive or negative basis, significantly more positive cases were seen on days 1–13 (61%) compared with days 14–28 (34%) ($P = 0.02$). However, taking the more quantitative approach to assessment in terms of OR scores (Table I), the range of positive values seen on comparing days 1–13 (8–406) and 14–28 (54–410) was similar; median scores did not differ significantly. Yet, the distribution of scores was not even through the cycle. To examine this in more detail an arbitrary cut-off score for high values was taken at 200, to give three levels, namely negative, low-moderate and high as displayed in Figure 3, which also details the number of cases included in each time period. This illustrated a highly significant decrease in the proportion of low to moderate scores in the second half of the cycle ($P = 0.0001$), with an equivalent increase in negative cases. The proportion of high scoring cases (≥200) stayed the same throughout.

Looking at OC use, only 26% of cases gave positive OR detection, although the range of positive OR scores (9–417) was similar to that in the natural cycle. Overall, reactivity was significantly less frequent than in the natural menstrual cycle, either assessed as a proportion of positive cases ($P = 0.005$) or as a score according to positive and negative cases ($P = 0.014$) (Tables I and II). The decrease in positive reactivity represented a significant decrease in both high ($P < 0.001$) and low to moderate ($P < 0.02$) scoring cases (Figure 3). In this group, no clear differences emerged for OR comparing days 1–13 with days 14–28 of the cycle (Tables I and II). There was a non-significant trend for more frequent OR detection in the week off OC.

*Effect of age and parity* The influence of age on OR detection was considered in two ways, either as chronological age (time since birth) or breast age (time since menarche). In neither instance was there a significant influence on OR immunoreactivity. This was true for both natural and artificial (OC) cycles. Similarly, comparing nulliparous and parous groups no differences were apparent in receptor detection in natural cycles or during OC use (Table I). Yet considering only parous women and in the natural cycle, the interval since last pregnancy had an effect on both the proportion of OR positive cases and levels of OR scores. For example, positive OR reactivity was noted in only two of 13 cases from women ≤2 years post partum (positive scores = 237 + 331). Expanding the cut-off to 5 years to give greater numbers for statistical analysis, positive cases were significantly more frequent ($P = 0.005$) and the median score was higher ($P = 0.0002$) in breast tissue from women whose last pregnancy was ≥5 years compared with those who had been pregnant within the last 5 years. From this type of subgroup analysis, it was not known whether the significant difference represented an increase in detection amongst those ≥5 years post partum or a decrease in those more recently parous. This problem was overcome by multivariate analysis (see below), which also allowed phases of menstrual cycle and OC use to be taken into account when considering age or parity.

### *Progesterone receptor detection*

*Effect of menstrual cycle type and day* In the natural cycle, more than 70% of cases showed positive immunoreactivity for PR. When cases were assessed either on a simple positive/negative basis or in terms of a PR score, no significant differences were seen comparing days 1–13 of the cycle with days 14–28 (Table I). Taking the same division at 200 as for OR, a large proportion of positive cases were in the high (≥200 group) (Figure 4). This reflected the arbitrary nature of the cut-off, which may have a different significance for PR than for OR.

Considering OC use, PR was detected with similar frequency to that seen in the natural cycle (>70%). For OC users, the median PR score was significantly higher on days 14–28 of the cycle compared with days 1–13 (Tables I and II). Looking across the entire OC cycle, there was a steady rise in high scoring PR positive cases (≥200) progressing from week 1 (days 1–5) to week 4 (21–28) (Figure 4), with a significant increase on days 14–28 compared with days 1–13 ($P < 0.05$). This increase was accompanied by a decrease in both negative and low to moderate cases.

*Effect of age and parity* As for OR, neither chronological age nor breast age influenced PR detection. However in the natural cycle, there was a non-significant trend ($P = 0.078$) for PR scores to be lower amongst parous women. As before, looking at the effect of interval since pregnancy in the natural cycle a low proportion (2/11) of women ≤2 years post partum showed positive PR reactivity (positive scores 412 + 233).

Adopting the same division as oestrogen, at 5 years the proportion of positive cases did not differ significantly. However, those who were parous within 5 years showed a significantly lower ($P = 0.009$) median PR score than those pregnant 5 or more years previously (Tables I and II). The effect is examined in more detail below by multivariate analysis.

### *Multivariate analysis*

Multivariate analysis was performed for cases scored on a simple positive or negative basis and findings are presented in Table III. Adjusting for phase of the menstrual cycle, OC use had a significant negative influence on the frequency of OR positivity. Breast age and parity did not influence the frequency of receptor positive detection, when adjusted for cycle phase and oral contraceptive use, but time since pregnancy was a highly significant influencing factor. Compared with nulliparous, those less than 5 years post partum were less frequently OR positive, while those 5–10 years following pregnancy showed a much higher frequency of detection.

For PR, phase of the menstrual cycle, OC use and breast age had no significant influence, yet the reduction in the

**Table I** Factors affecting oestrogen and progesterone receptor detection in normal breast

| Feature | Oestrogen receptor scores | | | | | | Progesterone receptor scores | | | | | |
|---|---|---|---|---|---|---|---|---|---|---|---|---|
| | Natural cycle | | | Artificial (OC) cycle | | | Natural cycle | | | Artificial (OC) cycle | | |
| | (Pos/Tot) | (%) | Scores[b] | (Pos/Tot) | (%) | Scores[b] | (Pos/Tot) | (%) | Scores[b] | (Pos/Tot) | (%) | Scores[b] |
| Phase-days 1–13 | 25/41[a] | 61 | 0-406,63 | 11/32 | 34 | 0-417,0 | 26/34 | 76 | 0-491,209 | 17/29 | 59 | 0-517,79[a] |
| Phase-days 14–28 | 15/44 | 34 | 0-410,0 | 6/36 | 17 | 0-367,0 | 25/35 | 71 | 0-600,200 | 26/32 | 81 | 0-583,267 |
| Chronologic age | | | | | | | | | | | | |
| ⩽25 years | 15/33 | 45 | 0-513,0 | 12/44 | 27 | 0-424,0 | 20/25 | 80 | 0-600,217 | 27/41 | 66 | 0-583,150 |
| >25 years | 27/55 | 49 | 0-467,0 | 6/26 | 23 | 0-436,0 | 31/45 | 69 | 0-444,200 | 18/22 | 82 | 0-408,209 |
| Breast age | | | | | | | | | | | | |
| ⩽10 years | 11/25 | 44 | 0-513,0 | 5/26 | 19 | 0-417,0 | 17/20 | 85 | 0-419,196 | 15/25 | 60 | 0-517,120 |
| >10 years | 31/62 | 50 | 0-467,4 | 10/38 | 26 | 0-436,0 | 34/49 | 34 | 0-500,200 | 26/33 | 79 | 0-583,192 |
| Parity | | | | | | | | | | | | |
| Nullipar | 20/47 | 43 | 0-513,0 | 10/49 | 20 | 0-436,0 | 29/37 | 78 | 0-600,240 | 33/44 | 75 | 0-583,186 |
| Parous | 22/41 | 54 | 0-406,63 | 8/21 | 38 | 0-424,0 | 22/33 | 67 | 0-417,162 | 12/19 | 63 | 0-417,136 |
| Interval since pregnancy | | | | | | | | | | | | |
| <5 years | 7/23[a] | 30 | 0-331,0[a] | 4/13 | 31 | 0-424,0 | 10/19 | 53 | 0-412,18[a] | 7/13 | 54 | 0-417,39 |
| ⩾5 years | 14/17 | 82 | 0-467,283 | 4/8 | 50 | 0-358,4.5 | 11/13 | 85 | 0-417,292 | 5/6 | 83 | 0-400,143 |
| Type of cycle | 42/88[a] | 48 | 0-513,0[a] | 18/70 | 26 | 0-436,0 | 51/70 | 73 | 0-600,200 | 45/63 | 71 | 0-583,179 |

[a]Significant differences, *P* values given in Table II. [b]Range, median.

**Table II** Levels of significance for comparisons in Table I

| | *Positive percentage* $\chi^2$*-test* | *Postive score (Mann-Whitney* U *test)* |
|---|---|---|
| *Oestrogen receptor* | | |
| Phase of natural cycle | 0.02 | NS |
| Type of cycle (nat *vs* OC) | 0.005 | 0.014 |
| Interval since pregnancy | 0.005 | 0.0002 |
| *Progesterone receptor* | | |
| Phase of OC cycle | NS | 0.05 |
| Type of cycle (nat *vs* OC) | NS | NS |
| Interval since pregnancy | NS | 0.0009 |

NS = not significant.

**Figure 3** Distribution of cases stained for oestrogen receptor according to proportion of negative (0), low-moderate (<200) and high (⩾200) scores across natural and artificial (OC) menstrual cycles. (●—●) Negative, (■—■) Low-mod, (▲—▲) High.

**Figure 4** Distribution of cases stained for progesterone receptor according to proportion of negative (0), low-moderate (<200) and high (⩾200) scores across natural and artifical (OC) menstrual cycles. (●—●) Negative, (■—■) Low-mod, (▲—▲) High.

frequency of PR positive cases among parous women approached significance (Table III). Time since last pregnancy did have an independent effect on the likelihood of PR positivity, in that there was a significant reduction in positive cases amongst those <5 years post partum compared with nulliparous individuals.

Looking at OC formulation, classified as described previously (Anderson *et al.*, 1989), after adjustment for phase of cycle, OC use and time since last pregnancy, there was no significant effect of oestrogen or progestogen content on proportion of positive cases.

**Table III** Multivariate analysis of factors affecting oestrogen and progesterone receptor detection

| | *Oestrogen receptor* | | | *Progesterone receptor* | | |
|---|---|---|---|---|---|---|
| | $\chi^2$ | *d.f.* | *P*< | $\chi^2$ | *d.f.* | *P*< |
| Phase of cycle | 11.95 | 4 | 0.05 | 2.64 | 4 | NS |
| Adjusting for phase | | | | | | |
| Current OC use | 8.80 | 1 | 0.01 | 0.10 | 1 | NS |
| Adjusting for OC use | | | | | | |
| Parity | 1.91 | 1 | NS | 3.70 | 1 | 0.10 |
| Interv since last preg | 17.84 | 2 | 0.0001 | 12.10 | 2 | 0.01 |
| Adjusting for time since last pregnancy | | | | | | |
| Breast age | 3.20 | 5 | NS | 2.02 | 2 | NS |
| Oestrogen content of OC | 2.80 | 4 | NS | 5.45 | 4 | NS |
| Progestogen content of OC | 2.70 | 5 | NS | 5.70 | 4 | NS |

A complicating factor for multivariate analysis of receptor scores was the high frequency of negative cases. However, taking only those cases postive, results could be analysed according to receptor scores. No effect on OR or PR level was seen for any of the parameters tested, yet it must be noted that omission of negatives made case numbers small.

### Discussion

This study agrees with previous reports of OR and PR reactivity in epithelial cell nuclei of normal breast tissue, commonly expressed as a low proportion of positive cells (Petersen *et al.*, 1987; Jacquemier *et al.*, 1990; Joyeux *et al.*, 1990). We now extend the observations through a semi-quantitative evaluation that acknowledges not only the functional integrity of TDLU as units of response, but also the heterogeneity of immunoreactive receptor presence, both within and between the units. Such factors have not previously been considered and provide a different perspective for interpretation of breast response.

For OR, the significant excess of cases with positive reactive tissues seen in the first half of the menstrual cycle has been noted previously both on biochemical (Silva *et al.*, 1983) and immunohistochemical (Williams *et al.*, 1991) assessment as well as on needle aspirated breast cells (Markopoulos *et al.*, 1988). Thus, with the addition of the present results, it would appear that the prominence of OR within the first half of the cycle is established. However, it must be noted that two immunohistochemical studies have shown no correlation between cycle phase and OR detection (Carpenter *et al.*, 1989; Jacquemier *et al.*, 1990). This may be because in the study of Carpenter *et al.* (1989), assignment of breast tissue to phases of cycle was based on histological grounds (Vogel *et al.*, 1981) rather than specific calendar dates, as in the present series, while the study of Jacquemier *et al.* (1990) was based on a series of only 15 cases.

The decrease in OR positive cases in the second half of the menstrual cycle is the same as the endometrium (Press *et al.*, 1984; Lessey *et al.*, 1988) and is consistent with the accepted dogma of OR down-regulation as a consequence of progesterone action. The present study further supports this from observations in OC cycle, where there is a striking decrease in detection with use, presumably on account of down-regulation by exogenous progestins. However, the present data do not enable an evaluation of any contribution to the down-regulation by oestrogen itself. The utility of the scoring system has been to demonstrate that there are two patterns of staining, one of which is constant in the natural cycle and the other which varies. Since the biopsy samples are solitary and not sequential, there are major limitations on the inferences to be drawn from these patterns in terms of epithelial response and activity.

The current literature on PR in normal breast epithelium is sparse, with only one report of 15 cases (Jacquemier *et al.*, 1990) indicating menstrual cycle variation, with an increase in the secretory phase. Two other reports (Joyeux *et al.*, 1990; Williams *et al.*, 1991) show no change through the cycle. Our data agree with the latter publications, although we cannot rule out a small, but statistically insignificant oestrogen-related increase in staining during the late proliferative stage of the natural cycle. Of considerable interest is the detection by the scoring system of a significant stimulatory effect of OC use on PR staining, with increasing days of use. This is difficult to interpret, but as most OC types used by women in this study contained oestrogen in addition to progestin, it is possible that the rise reflects an oestrogenic effect not counteracted by the progestin component. However, the absence of such an effect in the natural cycle is against that idea. Further anlaysis of epithelia in women taking progestin only OC may help to resolve the problem, but there is the caveat that significant ovarian follicular function occurs within this group (Howie, 1985). What is clear from our data is that neither endogenous (natural) or exogenous (OC) progestins dramatically down-regulate breast epithelial PR. This contrasts with endometrial receptors (Press *et al.*, 1988) and thus provides another example in which these two types of epithelium differ.

This study does not specifically address the issue of steriod receptor reactivity and proliferation, and thus no direct comment is offered upon the role, if any, of progesterone as such a breast stimulant. Nevertheless, the suggestion that progestins promote cell division is supported by studies of fatty acid synthetase, a marker of progesterone response (Chalbos *et al.*, 1987). This enzyme shows increased localisation in normal breast epithelium in the second half of the menstrual cycle (Joyeux *et al.*, 1990) and an association has been reported with proliferative varieties of benign breast lesions (Chalbos *et al.*, 1990). Yet *in vitro* studies with explants in nude mice have demonstrated a stimulant effect of oestrogen on normal breast epithelial proliferation, while progesterone has no effect (McManus & Welsch, 1984; Laidlaw *et al.*, 1990). Resolving these differences is challenging. As we have previously shown, there are several variables significantly influencing $^{3}H$ thymidine uptake in *ex vivo* normal breast (Anderson *et al.*, 1989). It thus appears unduly simplistic to suggest that proliferative activity in this tissue is controlled by either steroid acting alone. Indeed, it may be that the ratio of oestrogen to progesterone is critical in modulating the levels of receptor expressed and ultimately the level of proliferation, whether stimulated either directly or indirectly by steroid action or via other growth factors. However, our current data do not help to resolve the question of which female sex steroid hormones are the prime regulators of normal breast epithelial proliferation (King, 1990).

The present study of receptor variations during menstrual cycles has revealed several idiosyncratic features concerning breast responses that suggest approaches emphasising local factors should be followed. As in the case of proliferation (Anderson *et al.*, 1989), multivariate analysis of the present data has identified independent variables influencing steroid receptor expression, of which time since pregnancy shows a major effect. This is further evidence for an altered tissue environment following pregnancy, as already suggested from the decrease in proliferative response in breast tissue from parous OC users (Anderson *et al.*, 1989). It is also supported by the very low incidence of OR and PR detection seen in breast tissue from women $\leqslant 2$ years post-pregnancy. The pregnancy effect may be the result of variations in tissue hormonal environment or altered responsiveness to this environment. Certainly, there is evidence from nipple aspirates for a decrease in local oestrogen content of breast tissue among those less than 5 years post-pregnancy (Petrakis *et al.*, 1987). Alternatively, an explanation for altered steroid receptor reactivity can be based on histology, from the observation that, in a proportion of women following pregnancy, breast TDLU show a markedly atrophic morphological appearance recognisable up to 5 years post partum (Battersby & Anderson, 1989). This would readily account for refractory behaviour of TDLU to local endocrine factors. An understanding of the mechanisms by which these alterations are achieved and sustained may be crucial in explaining the decrease in breast cancer risk associated with an early pregnancy (MacMahon *et al.*, 1970).

The continued cooperation of the surgical and theatre staff, Department of Surgery, Longmore Hospital, Edinburgh, is gratefully acknowledged. We thank Mrs Lynda Ferrigan and Mrs Lorraine Allison for technical assistance, Dr James Going for help with sample collection and Mrs Joyce Garson for typing the manuscript.

The study was supported by the Imperial Cancer Research Fund.

### References

ANDERSON, T.J., BATTERSBY, S., KING, R.J.B., MCPHERSON, K. & GOING, J.J. (1989). Oral contraceptive use influences resting breast proliferation. *Human Pathol.*, **20**, 1139.

BATTERSBY, S. & ANDERSON, T.J. (1989). Histological changes in breast tissue that characterize recent pregnancy. *Histopathology*, **15**, 415.

CARPENTER, S., GEORGIADE, G., MCCARTY, K.S. Sr & MCCARTY, K.S. Jr (1989). Immunohistochemical expression of oestrogen receptor in normal breast tissue. *Proc. Roy. Soc. Edinb.*, **95B**, 59.

CHALBOS, D., CHAMBON, M., AILHAUD, C. & ROCHEFORT, H. (1987). Fatty acid synthetase and its mRNA are induced by progestins in breast cancer cells. *J. Biol. Chem.*, **262**, 9923.

CHALBOS, D., ESCOT, C., JOYEUX, C., TISSOT-CARAYON, M.-J., PAGES, A. & ROCHEFORT, H. (1990). Expression of the progestin-induced fatty acid synthetase in benign mastopathies and breast cancer as measured by RNA in situ hybridization. *J. Natl Cancer Inst.*, **82**, 602.

HELIN, H.J., HELLE, M.J., HELIN, M.L. & ISOLA, J.J. (1988). Immunocytochemical detection of estrogen and progesterone receptors in 124 human breast cancers. *Am. J. Clin. Pathol.*, **90**, 137.

HOWIE, P.W. (1985). The progestogen-only pill. *Br. J. Obstet. Gynaecol.*, **92**, 1001.

JACQUEMIER, J.D., HASSOUN, J., TORRENTE, M. & MARTIN, P.-M. (1990). Distribution of estrogen and progesterone receptors in healthy tissue adjacent to breast lesions at various stages – immunohistochemical study of 107 cases. *Breast Cancer Res. Treat.*, **15**, 109.

JOYEUX, C., CHALBOS, D. & ROCHEFORT, H. (1990). Effects of progestins and menstrual cycle on fatty acid synthetase and progesterone receptor in human mammary glands. *J. Clin. Endocrinol. Metab.*, **70**, 1438.

KING, R.J.B. (1990). Role of oestrogen and progestin in human mammary carcinogenesis. In *Endocrine Therapy of Breast Cancer IV*, Goldhirsch, A. (ed.), p. 3. Springer-Verlag: Berlin.

LAIDLAW, I.J., CLARKE, R., ANDERSON, E. & HOWELL, A. (1990). Proliferation of normal human breast tissue in nude mice after ovarian hormone stimulation. *Br. J. Surg.*, **77**, A1419.

LESSEY, B.A., KILLAM, A.P., METZGER, D.A., HANEY, A.F., GREENE, G.L. & MCCARTY, K.S. Jr (1988). Immunohistochemical analysis of human uterine estrogen and progesterone receptors throughout the menstrual cycle. *J. Clin. Endocrinol. Metab.*, **67**, 334.

MARKOPOULOS, C., BERGER, U., WILSON, P., GAZET, J.-C. & COOMBES, R.C. (1988). Oestrogen receptor content of normal breast cells and breast carcinomas throughout the menstrual cycle. *Br. Med. J.*, **296**, 1349.

MCCARTY, K.S. Jr (1989). Proliferative stimuli in the normal breast: estrogens or progestins? *Human Pathol.*, **20**, 1137.

MCCARTY, K.S., Jr, MILLER, L.S., COX, E.B., KONRATH, J. & MCCARTY, K.S. Sr (1985). Estrogen receptor analyses. Correlation of biochemical and immunohistochemical methods using monoclonal antireceptor antibodies. *Arch. Pathol. Lab. Med.*, **109**, 716.

MACMAHON, B., COLE, P., LIN, T.M. & 6 others (1970). Age at first birth and breast cancer risk. *Bull WHO*, **43**, 209.

MCMANUS, M.J. & WELSCH, C.W. (1984). The effect of estrogen, progesterone, thyroxine and human placental lactogen on DNA synthesis of human breast ductal epithelium in athymic nude mice. *Cancer*, **54**, 1920.

PATERSON, D.A., REID, C.P., ANDERSON, T.J. & HAWKINS, R.A. (1990). Assessment of oestrogen receptor content of breast carcinoma by immunohistochemical techniques on fixed and frozen tissue and by biochemical ligand binding assay. *J. Clin. Pathol.*, **43**, 46.

PETRAKIS, N.L., WRENSCH, M.R., ERNDER, V.L. & 4 others (1987). Influence of pregnancy and lactation on serum and breast fluid estrogen levels: implications for breast cancer risk. *Int. J. Cancer*, **40**, 587.

PETERSEN, O.W., HOYER, P.E. & VAN DEURS, B. (1987). Frequency and distribution of estrogen receptor-positive cells in normal nonlactating human breast tissue. *Cancer Res.*, **47**, 5748.

PRESS, M.F., NOUSEK-GOEBL, N.A., KING, W.J., HERBST, A.L. & GREENE, G.L. (1984). Immunohistochemical assessment of estrogen receptor distribution in the human endometrium throughout the menstrual cycle. *Lab. Invest.*, **51**, 495.

PRESS, M.F., UNDOVE, J.A. & GREENE, G.L. (1988). Progesterone receptor distribution in the human endometrium. *Endocrinology*, **122**, 1165.

SHINTAKU, P. & SAID, J.W. (1987). Detection of estrogen receptors with monoclonal antibodies in routinely processed formalin-fixed paraffin section of breast carcinoma. *Am. J. Clin. Pathol.*, **87**, 161.

SILVA, J.S., GEORGIADE, G.S., DILLEY, W.G., MCCARTY, K.S. Sr, WELLS, S.A. Sr & MCCARTY, K.S. Jr (1983). Menstrual cycle-dependent variations of breast cyst fluid proteins and sex steroid receptors in the normal human breast. *Cancer*, **51**, 1297.

STEFANINI, M., DE MARTINO, C. & ZAMBONI, L. (1967). Fixation of ejaculated spermatozoa for electron microscopy. *Nature*, **215**, 173.

THOMAS, D.B. (1991). Oral contraceptives and breast cancer: review of the epidemiologic literature. *Contraception*, **43**, 597.

VOGEL, P.M., GEORGIADE, N.G., FETTER, B.J., VOGEL, F.S. & MCCARTY, K.S. Jr (1981). The correlation of histological changes in the human breast with the menstrual cycle. *Am. J. Pathol.*, **104**, 23.

WILLIAMS, G., ANDERSON, E., HOWELL, A. & 4 others (1991). Oral contraceptive (OCP) use increases proliferation and decreases oestrogen receptor content of epithelial cells in the normal breast. *Int. J. Cancer*, **48**, 206.


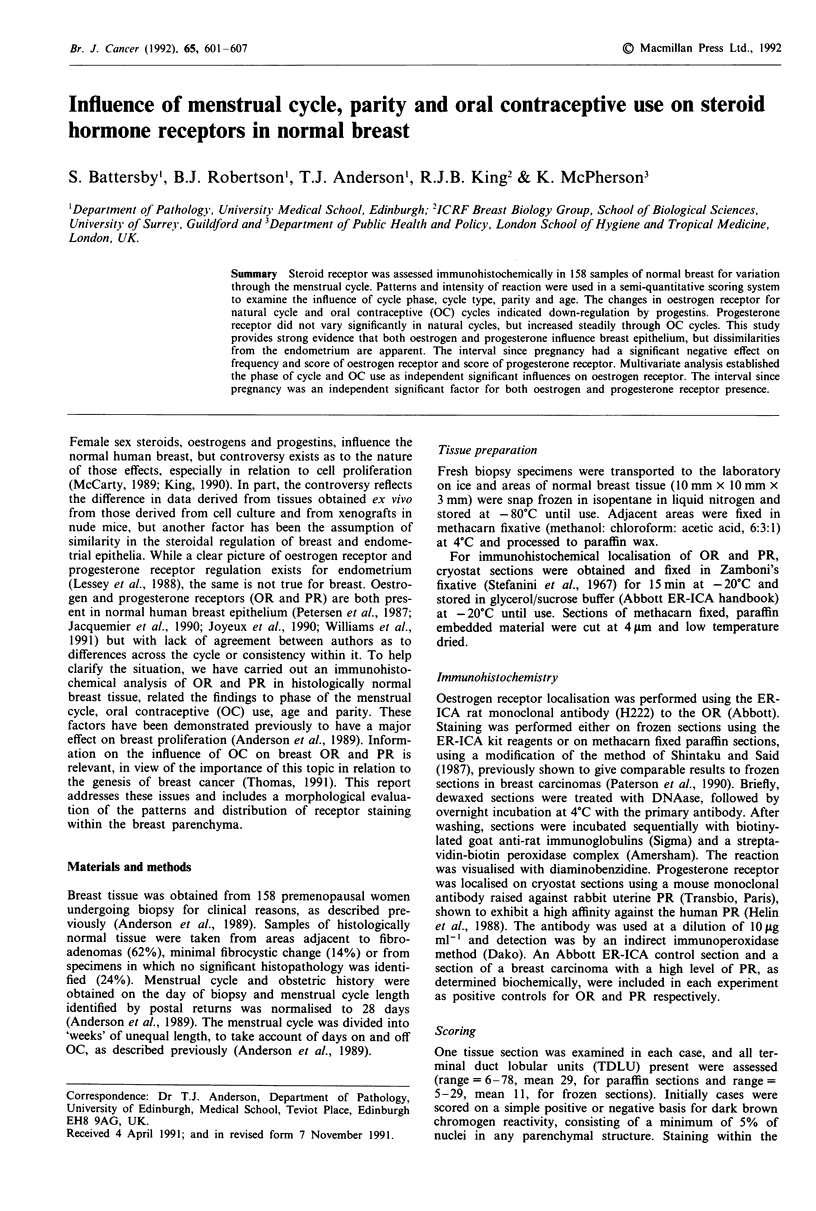

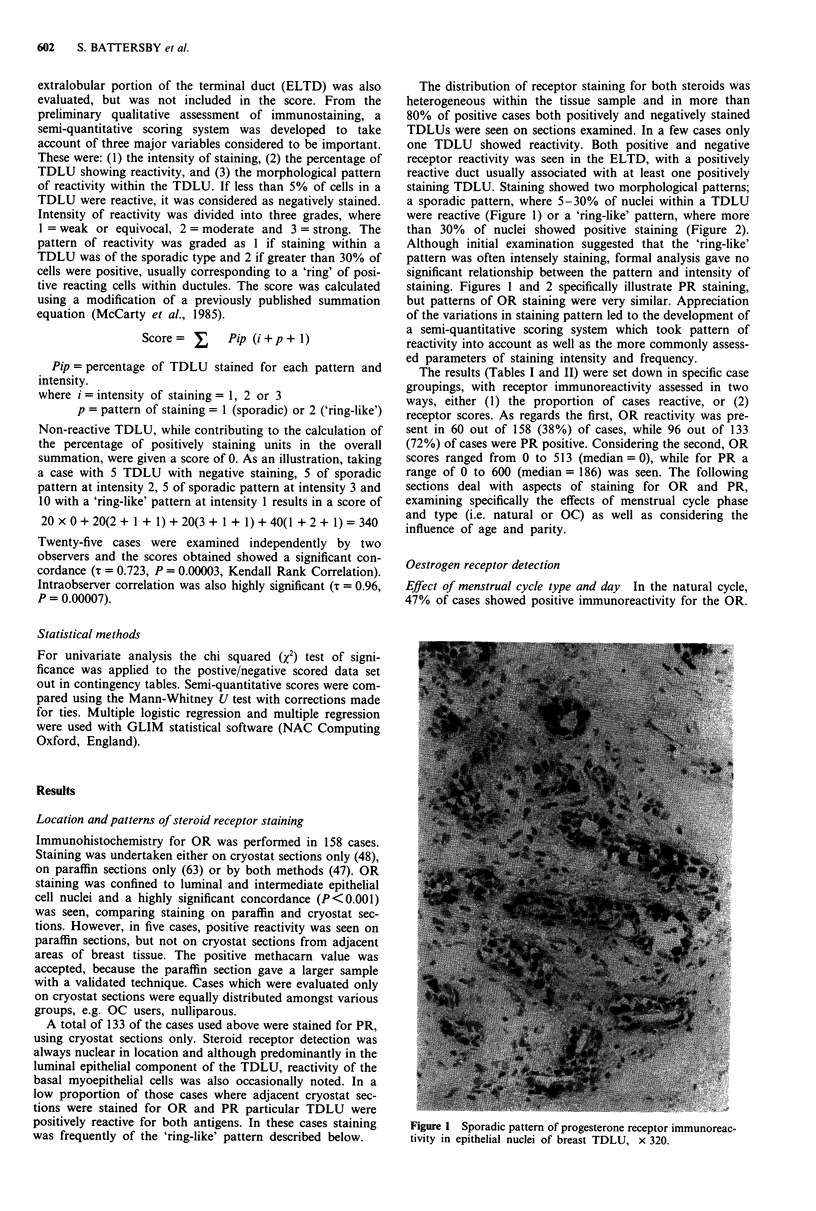

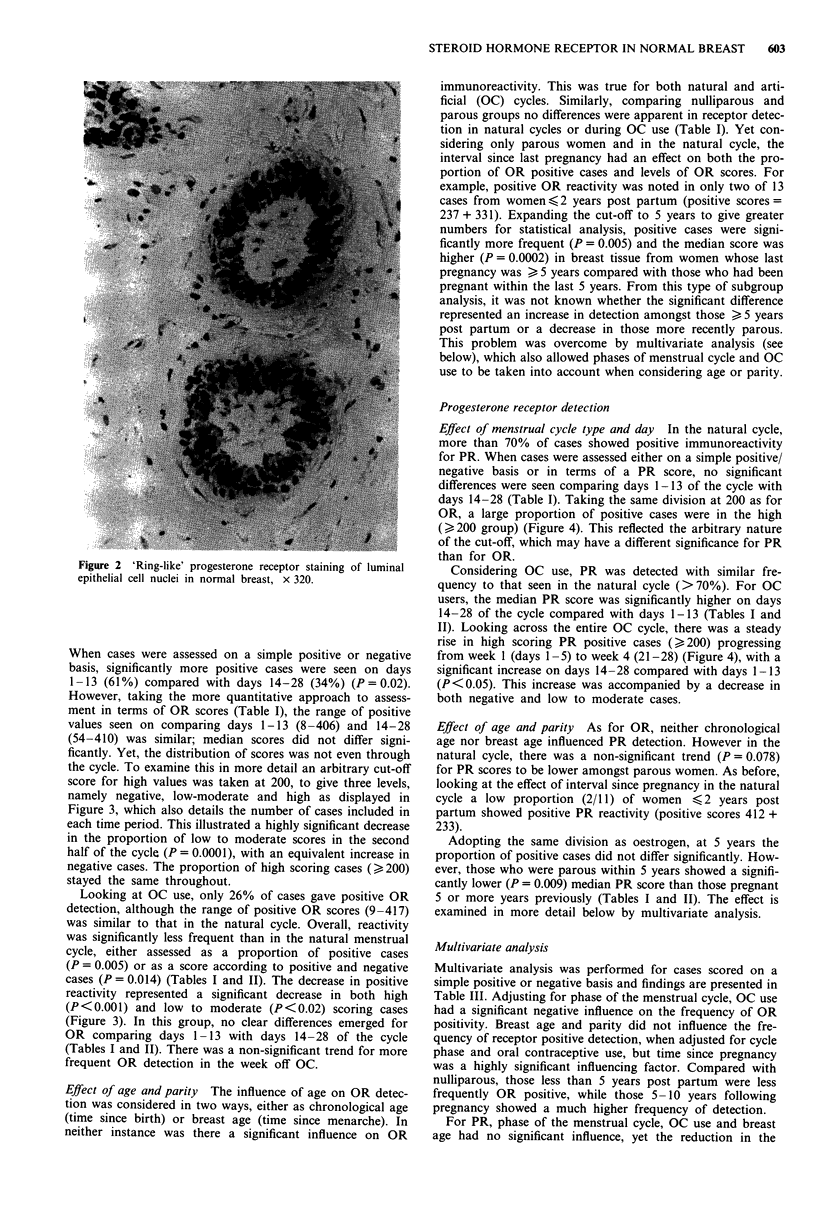

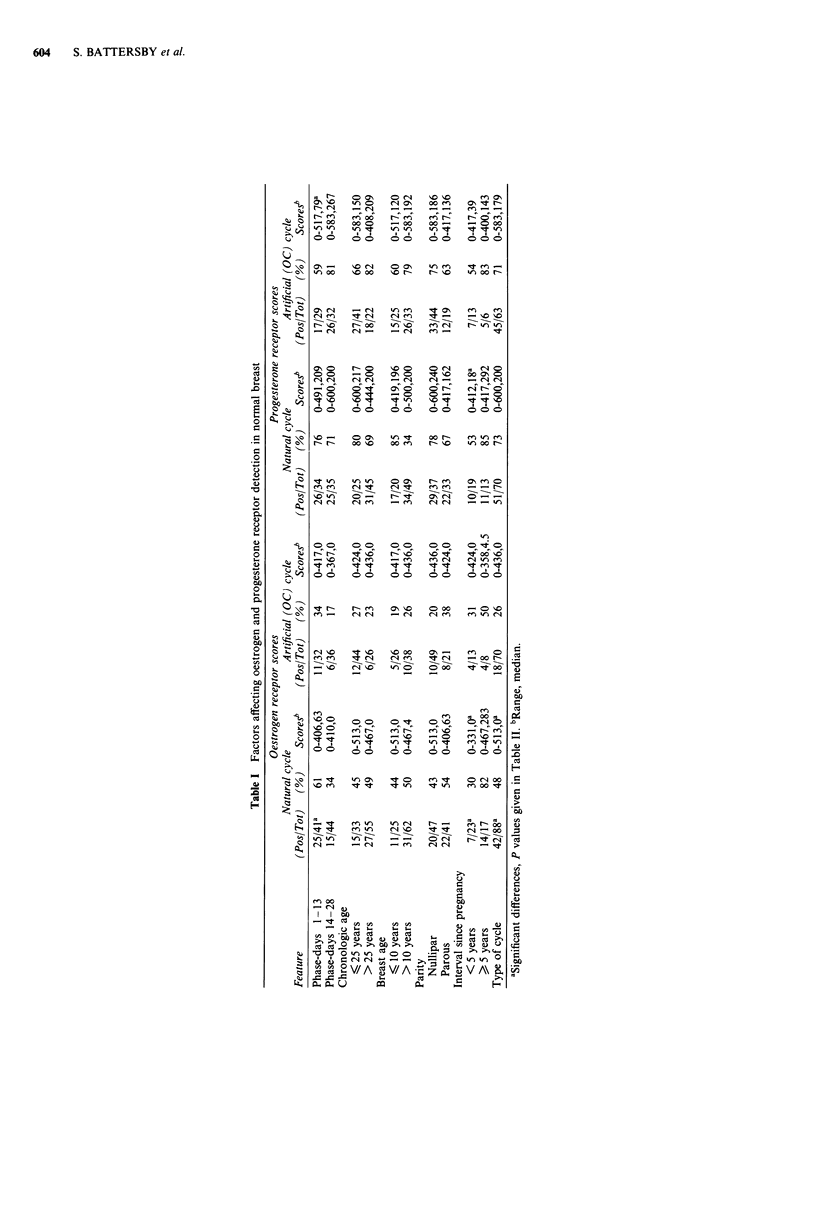

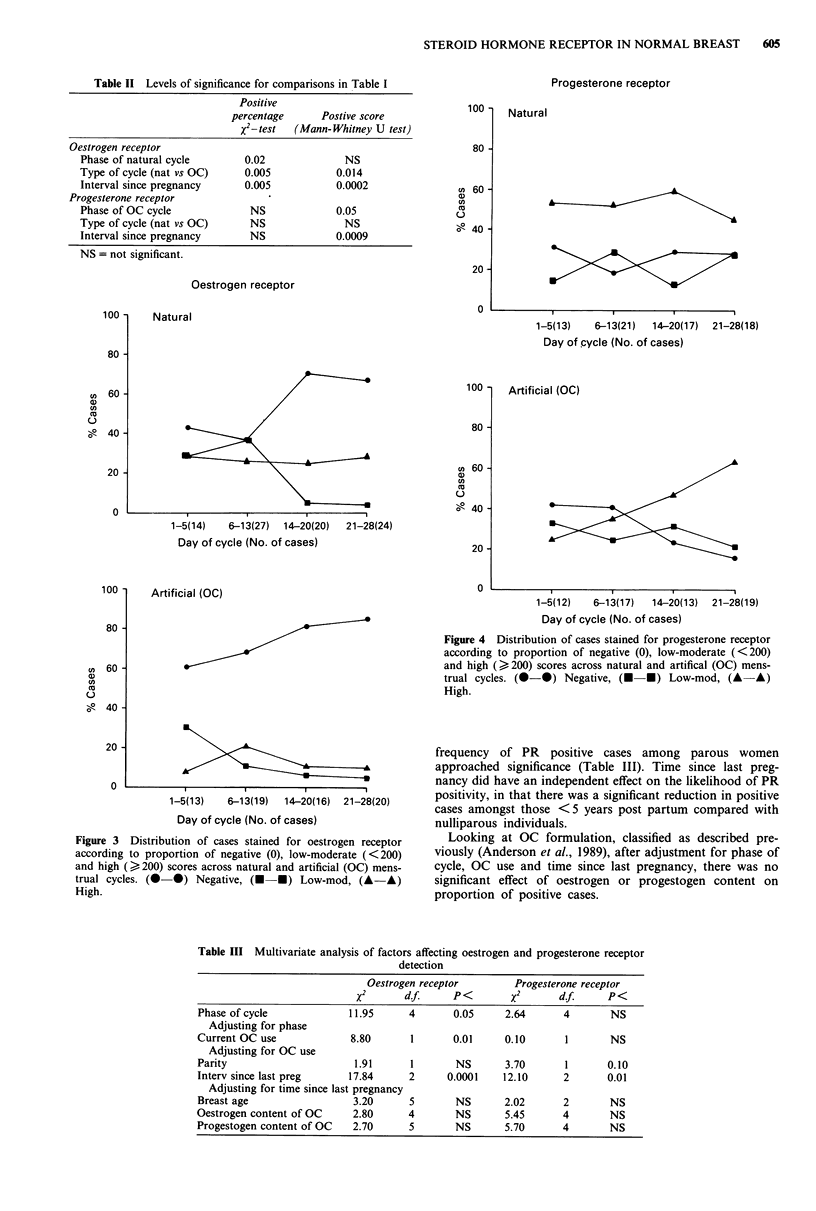

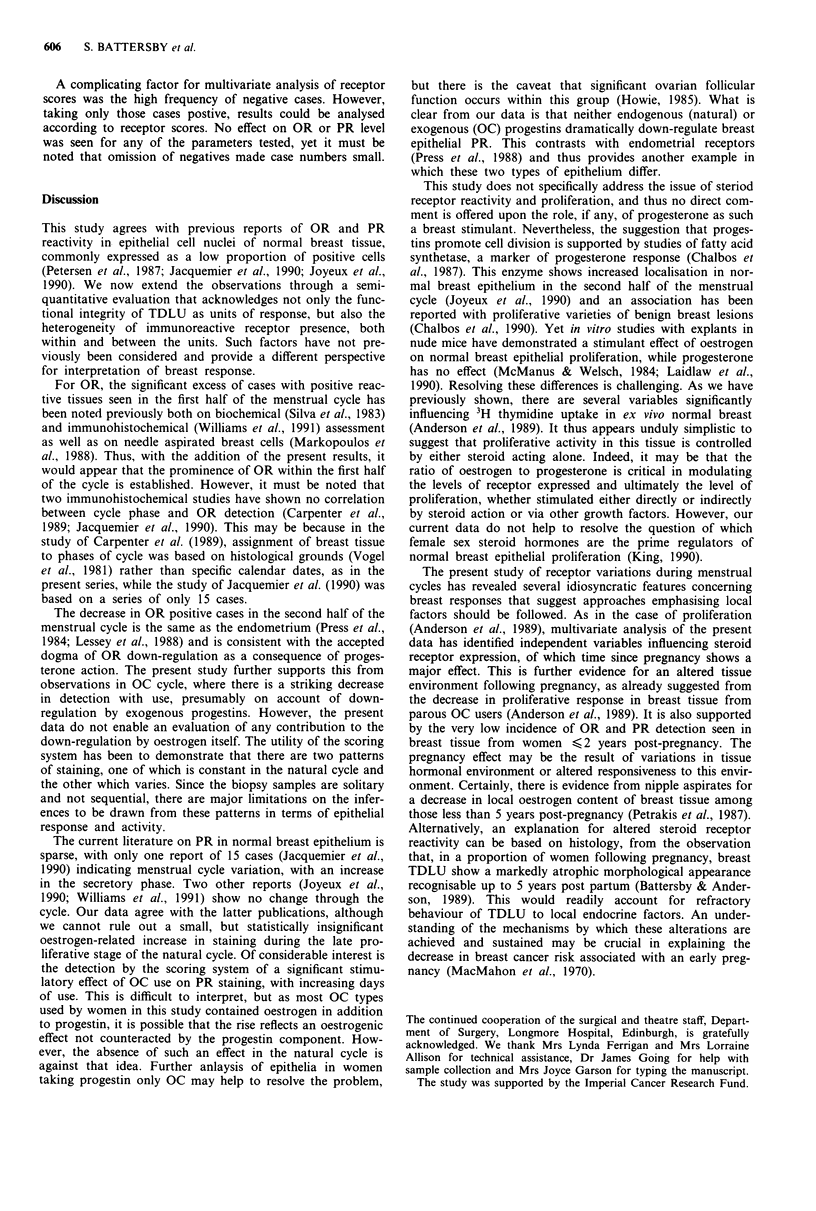

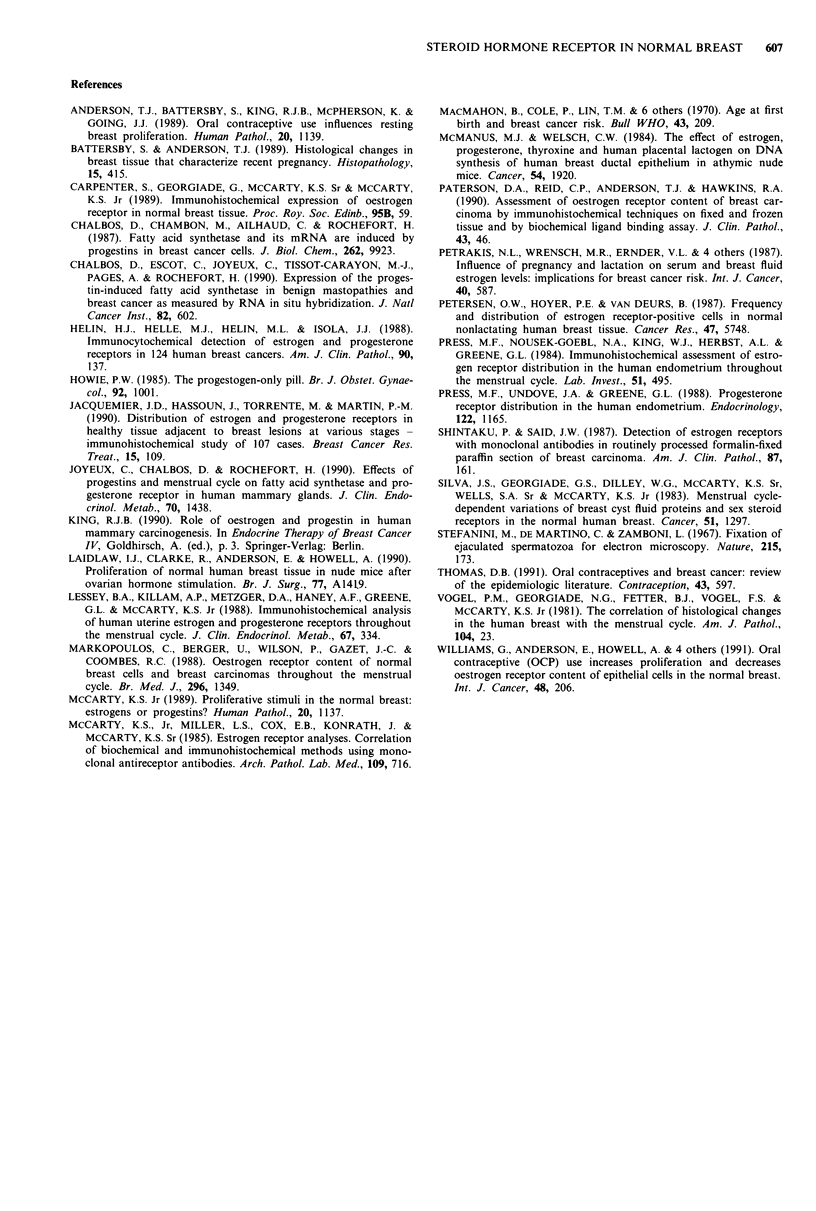


## References

[OCR_00966] Anderson T. J., Battersby S., King R. J., McPherson K., Going J. J. (1989). Oral contraceptive use influences resting breast proliferation.. Hum Pathol.

[OCR_00971] Battersby S., Anderson T. J. (1989). Histological changes in breast tissue that characterize recent pregnancy.. Histopathology.

[OCR_00980] Chalbos D., Chambon M., Ailhaud G., Rochefort H. (1987). Fatty acid synthetase and its mRNA are induced by progestins in breast cancer cells.. J Biol Chem.

[OCR_00985] Chalbos D., Escot C., Joyeux C., Tissot-Carayon M. J., Pages A., Rochefort H. (1990). Expression of the progestin-induced fatty acid synthetase in benign mastopathies and breast cancer as measured by RNA in situ hybridization.. J Natl Cancer Inst.

[OCR_00992] Helin H. J., Helle M. J., Helin M. L., Isola J. J. (1988). Immunocytochemical detection of estrogen and progesterone receptors in 124 human breast cancers.. Am J Clin Pathol.

[OCR_00998] Howie P. W. (1985). The progestogen-only pill.. Br J Obstet Gynaecol.

[OCR_01002] Jacquemier J. D., Hassoun J., Torrente M., Martin P. M. (1990). Distribution of estrogen and progesterone receptors in healthy tissue adjacent to breast lesions at various stages--immunohistochemical study of 107 cases.. Breast Cancer Res Treat.

[OCR_01009] Joyeux C., Chalbos D., Rochefort H. (1990). Effects of progestins and menstrual cycle on fatty acid synthetase and progesterone receptor in human mammary glands.. J Clin Endocrinol Metab.

[OCR_01025] Lessey B. A., Killam A. P., Metzger D. A., Haney A. F., Greene G. L., McCarty K. S. (1988). Immunohistochemical analysis of human uterine estrogen and progesterone receptors throughout the menstrual cycle.. J Clin Endocrinol Metab.

[OCR_01047] MacMahon B., Cole P., Lin T. M., Lowe C. R., Mirra A. P., Ravnihar B., Salber E. J., Valaoras V. G., Yuasa S. (1970). Age at first birth and breast cancer risk.. Bull World Health Organ.

[OCR_01031] Markopoulos C., Berger U., Wilson P., Gazet J. C., Coombes R. C. (1988). Oestrogen receptor content of normal breast cells and breast carcinomas throughout the menstrual cycle.. Br Med J (Clin Res Ed).

[OCR_01041] McCarty K. S., Miller L. S., Cox E. B., Konrath J., McCarty K. S. (1985). Estrogen receptor analyses. Correlation of biochemical and immunohistochemical methods using monoclonal antireceptor antibodies.. Arch Pathol Lab Med.

[OCR_01037] McCarty K. S. (1989). Proliferative stimuli in the normal breast: estrogens or progestins?. Hum Pathol.

[OCR_01051] McManus M. J., Welsch C. W. (1984). The effect of estrogen, progesterone, thyroxine, and human placental lactogen on DNA synthesis of human breast ductal epithelium maintained in athymic nude mice.. Cancer.

[OCR_01057] Paterson D. A., Reid C. P., Anderson T. J., Hawkins R. A. (1990). Assessment of oestrogen receptor content of breast carcinoma by immunohistochemical techniques on fixed and frozen tissue and by biochemical ligand binding assay.. J Clin Pathol.

[OCR_01070] Petersen O. W., Høyer P. E., van Deurs B. (1987). Frequency and distribution of estrogen receptor-positive cells in normal, nonlactating human breast tissue.. Cancer Res.

[OCR_01064] Petrakis N. L., Wrensch M. R., Ernster V. L., Miike R., Murai J., Simberg N., Siiteri P. K. (1987). Influence of pregnancy and lactation on serum and breast fluid estrogen levels: implications for breast cancer risk.. Int J Cancer.

[OCR_01081] Press M. F., Greene G. L. (1988). Localization of progesterone receptor with monoclonal antibodies to the human progestin receptor.. Endocrinology.

[OCR_01075] Press M. F., Nousek-Goebl N., King W. J., Herbst A. L., Greene G. L. (1984). Immunohistochemical assessment of estrogen receptor distribution in the human endometrium throughout the menstrual cycle.. Lab Invest.

[OCR_01086] Shintaku I. P., Said J. W. (1987). Detection of estrogen receptors with monoclonal antibodies in routinely processed formalin-fixed paraffin sections of breast carcinoma. Use of DNase pretreatment to enhance sensitivity of the reaction.. Am J Clin Pathol.

[OCR_01092] Silva J. S., Georgiade G. S., Dilley W. G., McCarty K. S., Wells S. A., McCarty K. S. (1983). Menstrual cycle-dependent variations of breast cyst fluid proteins and sex steroid receptors in the normal human breast.. Cancer.

[OCR_01098] Stefanini M., De Martino C., Zamboni L. (1967). Fixation of ejaculated spermatozoa for electron microscopy.. Nature.

[OCR_01103] Thomas D. B. (1991). Oral contraceptives and breast cancer: review of the epidemiologic literature.. Contraception.

[OCR_01107] Vogel P. M., Georgiade N. G., Fetter B. F., Vogel F. S., McCarty K. S. (1981). The correlation of histologic changes in the human breast with the menstrual cycle.. Am J Pathol.

[OCR_01113] Williams G., Anderson E., Howell A., Watson R., Coyne J., Roberts S. A., Potten C. S. (1991). Oral contraceptive (OCP) use increases proliferation and decreases oestrogen receptor content of epithelial cells in the normal human breast.. Int J Cancer.

